# Core strength: A new model for injury prediction and prevention

**DOI:** 10.1186/1745-6673-2-3

**Published:** 2007-04-11

**Authors:** WF Peate, Gerry Bates, Karen Lunda, Smitha Francis, Kristen Bellamy

**Affiliations:** 1 University of Arizona, Mel and Enid Zuckerman Arizona College of Public Health, Drachman Hall, 1295 N. Martin Avenue, Tucson, Arizona, USA; 2 Tucson Fire Department, Health and Safety, 421 South Church, Tucson, Arizona, USA; 3 Lunda and Associates, 1636 North Swan, Tucson, Arizona, USA

## Abstract

**Objective:**

Many work in injury prone awkward positions that require adequate flexibility and strength in trunk stabilizer muscle groups. Performance on a functional movement screen (FMS) that assessed those factors was conducted and an intervention was designed.

**Methods:**

A battery of FMS tests were performed on 433 firefighters. We analyzed the correlation between FMS performance and injuries and other selected parameters. An intervention to improve flexibility and strength in trunk stabilizer or core muscle groups through a training program was evaluated.

**Results:**

The intervention reduced lost time due to injuries by 62% and the number of injuries by 42% over a twelve month period as compared to a historical control group.

**Conclusion:**

These findings suggest that core strength and functional movement enhancement programs to prevent injuries in workers whose work involves awkward positions is warranted.

## Background

The National Occupational Research Agenda (NORA) has identified traumatic injury and intervention effectiveness as two of its priority research areas. Injuries are the leading cause of mortality and loss of potential years of life for working individuals. This study focused on a unique method of injury prediction and prevention in high risk workers using a functional movement screen and core strength intervention [1].

Many workers must deal with physically demanding tasks that involve awkward positions and less than optimal ergonomics. Fire fighting is a particularly hazardous profession with exposure to a host of chemical, biologic, and physical hazards including musculoskeletal trauma. Firefighters perform physically demanding tasks such as forcible entry and rescues that are injury prone because of maneuvers that compromise trunk stability and ergonomically hazardous conditions Because of the nature of fire fighting, these physical conditions are often difficult to control.

There are over one million fire fighters in the United States [2]. and the injury rates of firefighters are among the highest in all occupations [3]. Last year in the U.S. firefighters sustained 88, 500 injuries while on duty [4]. Forty four percent of all U.S. firefighters have suffered from sprains and strains while on duty [5].

It is important for firefighters to be fit because they work in physically unpredictable settings, and must maintain a high level of fitness for at least 20 years before they are eligible for retirement. Various strategies have been evaluated to decrease the occurrence and the severity of fire fighter injuries. These methods have focused on exercise training, ergonomic coaching and flexibility improvements [6]. A physical fitness intervention for firefighters was shown to be effective in reducing injuries, but the scope of the study was limited to back disorders [7]. A firefighter flexibility training program did not find improvement in injury incidence, though lost time, severity and costs improved [8]. Workplace injuries are multi-factorial, especially in occupations where work events are unpredictable and task completion places rigorous demands on the body. Furthermore, many ergonomic interventions have limited applicability in certain firefighter tasks. For example, a firefighter who must crawl under wreckage and contort his or her body to rapidly rescue a trapped individual has severe ergonomic challenges that are difficult to address with standard ergonomic suggestions such as "lift with your legs, not your back." Although many firefighter exercise programs have focused on upper and lower body strength, they have paid less attention to core stability and strength (provided by spine stabilizers such as the transversus and multifidi muscles) and the other dimensions of movement that might decrease the chance of injury in the above scenario [9]. As Wilson et al summarize: "Core stability is the ability of the lumbopelvic hip complex to prevent buckling and to return to equilibrium after perturbation. Although static elements (bone and soft tissue) contribute to some degree, core stability is predominantly maintained by the dynamic function of muscular elements. There is a clear relationship between trunk muscle activity and lower extremity movement" [10].

Current research suggests that decreased core strength may contribute to injuries of the back and extremities, that training may decrease musculoskeletal damage, and that core stability can be tested using functional movement methods [11-13].

The purpose of this study was to explore methods to better assess the risk of firefighter injury due to functional movement performance, and to decrease injuries by using that information. The magnitude of injuries among firefighters warrant efforts to develop and assess the effectiveness of interventions. One approach has been to examine the relationship between simulated firefighting tasks and physical performance or functional measures [14]. Researchers have demonstrated that activities such as stair climbing ability are related to certain functional measures such as standing balance, reaction time, isometric muscle strength [15].

Furthermore, improvements in core or static strength, flexibility and the three dimensions of movement: acceleration; deceleration; and dynamic stabilization (the ability to maintain a stable posture while moving) have been proposed as additional injury prevention possibilities for fire fighters [16].

Our research objective was to determine whether results of measurement of functional movement were associated with a history of previous work-related injuries in this high risk population and to conduct an intervention. Functional movement screens were initially used to evaluate and rehabilitate patients with neuromuscular coordination issues, such as those with stroke or spinal trauma [17,18]. More recently, functional movement screens have been employed to assess the movement patterns of athletes. Those with a lower performance score have been found to be more likely to sustain an injury [19].

We used the functional movement screen (FMS) for fire fighters because their job tasks often require maximal physical performance, [20] thus making them "industrial" athletes. The relation between the FMS score and age, rank, tenure and gender was also assessed. If a correlation existed between functional movement screen performance and injuries, then appropriate interventions such as flexibility and core strength training could be initiated to decrease fire fighter injury rates. A second arm of the study involved a twelve month prospective analysis of such an intervention.

The functional movement screen consists of seven different functional movements that assess: trunk or core strength and stability; neuromuscular coordination; symmetry of movement; flexibility; acceleration; deceleration; and dynamic stability. Each of these seven movements corresponds to a firefighter activity. For example, one of the FMS measures is the rotatory stability test. This test requires the firefighter to maintain spinal column stability with upper and lower trunk motion while balancing their weight with one hand and knee on the floor. The maneuver duplicates the fire fighter work practice of staying low to the floor while entering a burning building (Heat rises. Standing subjects the fire fighter to higher thermal energy.). The other FMS tests and their correspondence to fire fighter essential functions include:

Hurdle step: body mechanics while stepping over an obstacle during a fire or rescue.

In-lunge movement: ability to take one long step forward and lunge downward, such a while using an axe to open a door during a fire.

Shoulder mobility: firefighter lifting and placing a SCBA (self contained breathing apparatus) respirator on their back.

Stability push-ups (press-ups):core strength while reaching through or around an obstruction during a fire or rescue.

Deep squat: ability to squat to avoid an overhead hazard during a fire or rescue.

Active straight leg raise: flexibility of the lumbar-pelvic complex and lower extremity muscles. Maintenance of torso and pelvic stability during awkward positions at a fire or rescue operation.

## Methods

### Subjects

Environment Occupational Health (EOH) Unit faculty of the University of Arizona were awarded a contract to provide medical surveillance, and injury prevention and treatment for Tucson Fire Department, an urban fire fighting agency in a community of 765, 000. All 433 subjects were involved in fire suppression activities and were on a full duty status. Age at time of the study ranged from 21 to 60 years with a mean of 41.8 years for males and 37.4 years for females. The subjects were 408 male (94.2 percent) and 25 female (5.8 percent).

Demonstration of the FMS tests was conducted by a trained fitness coordinator. Informed consent was provided by a fire department representative.

Scores on the seven FMS tests were based on the firefighter's ability to perform the respective test. Zero to three points were possible for each of the seven tests (Total of 21 points). The maximum number of points was given if the individual could fully perform the test without limitation of movement or pain. Lesser points were given for partial completion of the test and no points for failure to complete any elements of the test.

The battery of FMS tests were performed on 433 firefighters over a four week period in late 2004. We analyzed the correlation between FMS performance and a history of prior musculoskeletal injury from the fire department database, and other selected parameters (age, gender, tenure and rank).

One firefighter sustained a minor strain during the testing process, and fully recovered one week later and was returned to full duty.

**Figure 1:**
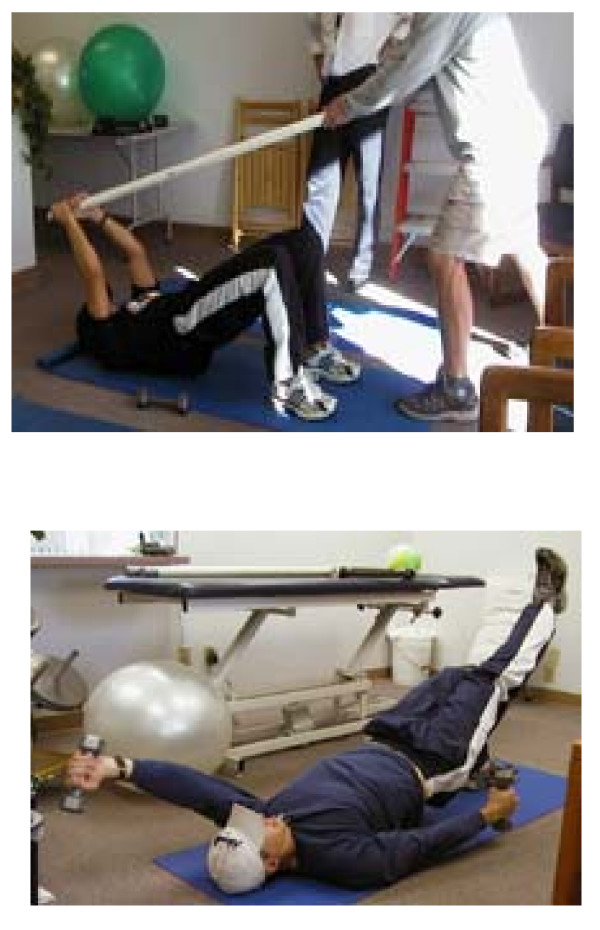
Keeping the transversus abdominus contracted and using the upper and lower extremities.

The firefighters were then enrolled in a training program designed by a multi-disciplinary team (occupational medicine physician, therapist, and fire department health and safety officer). Twenty one seminars, each three hours in length were conducted for groups of 20 firefighters over a two month period. Each session emphasized functional movement including the causation (inadequate core or back stabilizing muscle strength, poor flexibility, and improper body mechanics) and prevention of injuries. As part of the training session, each firefighter then demonstrated competency in the proper body mechanics in sample firefighter work settings. Firefighters are compelled to work in injury prone situations such as bending forward at the waist and reaching through the broken window of a wrecked automobile while assessing a victim. In this scenario core stabilizing muscles become fatigued and are at risk of injury. Participants were taught techniques to strengthen core muscles and to decrease mechanical load on the affected parts of their musculoskeletal system during these ergonomically challenging job tasks. For example, firefighters were instructed to use an outstretched arm held against a firm surface as a prop to decrease mechanical load on the back when the firefighter's spine is in lumbar flexion. Firefighters were instructed how to analyze the worksite and to use principles of functional movement (how to adjust to the employee's range of motion by moving closer to object to be lifted, to use postural relief or props, and "tighten the gut" or recruit stabilizing muscle before lifting). During each session, guidance and practice on core or stabilizing muscle strength exercises were offered. Demonstration of the exercises was provided by a trained co-worker. Core strength instructions were provided to each participant. They were advised to maintain a neutral position of the lumbar spine and to contract the transversus abdominus (TA) muscle. Participants were shown that muscle's location in the anterior abdominal wall. Photos of various methods of recruiting and strengthening the TA with written explanations were provided, along with verbal reinforcement of the material. Once the firefighter demonstrated competency in basic TA muscle tightening, physiotherapy balls and dowels were employed to challenge the firefighter in different positions that mimicked firefighting tasks. See Figure 1

- Lie on your back, knees bent, feet flat on the floor.

- Tighten the gut to maintain a neutral position of the low back (no arching or flattening)

- Lift up butt. Knees, hips and shoulders should all be in a line.

- If the butt starts sagging, lift it back up. If the hamstrings cramp, take a break and begin again.

- Add arm movement, one or both with or without weight/resistance

- Keep the butt up and the gut tight throughout the exercise.

- 5–10 reps, 1–3 sets of each variation of the exercise.

- Progress to a one legged bridge.

- Assume position above, lift the right foot up off of the floor.

- Extend knee out away from you (straighten the knee) and then bring it back toward you.

- Repeat the bending and straightening of the knee/leg

- The straighter and lower the leg, the harder the exercise.

- Keep the butt up and the gut tight throughout the exercise.

- Repeat with left leg off the floor.

- Add arm movement to leg movement and then add weights/resistance.

- 5–10 repetitions, 1–3 sets of each variation of the exercise.

See Figure 2.

**Figure 2:**
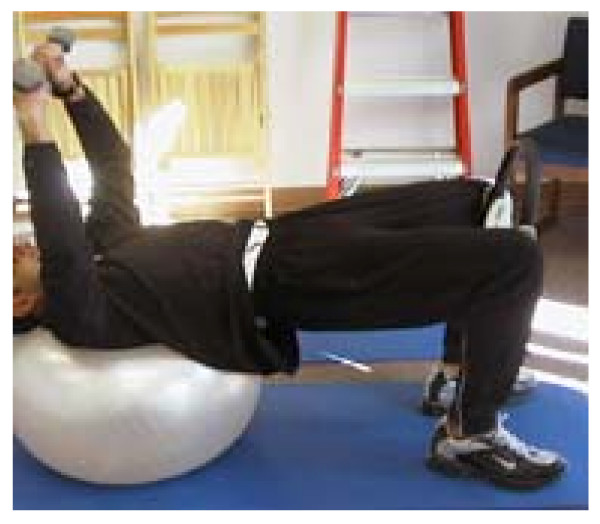
Bridging with shoulders on a ball.

- Correct physio-ball size equals a 90 degree knee bend when sitting on the ball. If greater than 90, inflate the ball. This does not need to be exact.

- Assume the starting position with shoulders on the ball, feet on the floor, knees bent to 90.

- The more of the back that is on the ball, the more stable, the easier the exercise.

- Shoulders, hips and knees in a line

- Tighten the gut

- Add arm movement, one or both with or without weights/resistance

- Do not let the back arch or flatten.

- To increase the difficulty, add a small object between the knees and squeeze or add a band around the knees and push the knees apart

- Keep the gut tight and the butt up

- To further increase the difficulty, roll further off of the ball so only the shoulders are on the ball.

- Perform 5 – 10 repetitions, 1 – 3 sets of each variation of the exercise

Figure 3

**Figure 3:**
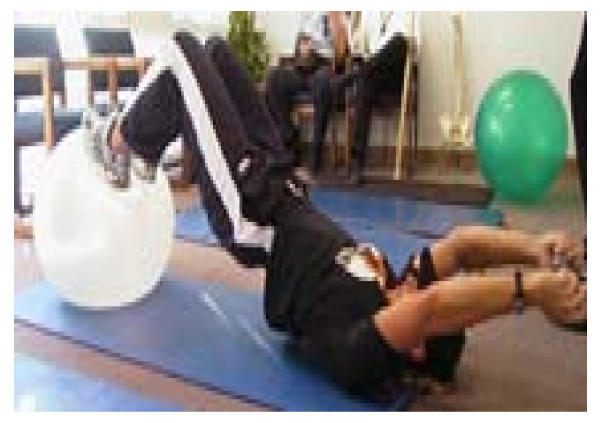
Bridging with feet on the ball.

- Correct physio-ball size equals a 90 degree knee bend when sitting on the ball. If greater than 90, inflate the ball. This does not need to be exact.

- Lye on your back, knees bent, soles of feet on the ball.

- Tighten your gut to maintain a neutral lumbar spine (no arching or flattening)

- Lift up your butt.

- If your butt starts sagging, lift it back up. If you can't, the set is over.

- If the hamstrings cramp, take a break and begin again.

- Arms may need to provide support/stability on the ground initially.

- Once stable, add arm movement, one or both with or without weight/resistance

- Keep the butt up and the gut tight.

- The further the arms go overhead, the more the back wants to arch.

- Prevent the arch by keeping the gut tight.

- If the back continues to arch, decrease the amount of arm movement or decrease the weight/resistance until you can maintain a neutral spine (no arching or flattening of the back).

- 5–10 reps, 1–3 sets of each variation of the exercise

For one year following training, information on the type and number of injury cases, cost of treatment, and lost days due to injury were gathered by the organization's worker's compensation department. The data was derived from personnel, absentee and medical records for a one-year period.

### Statistical Analyses

#### Part One. Functional Movement Screen

Data was coded using Stata 8.0. For exploratory data analysis we used bivariate methods. The primary hypothesis was assessed with multivariate analysis (logistic and linear regression). Table 1 provides functional movement screen summary descriptive statistics by overall score

**Table 1:** Summary Descriptive Statistics by Overall Score

**Score**	**Pass**	**Fail**	
	17	<17	
Count	300	133	
Percent	69.30%	30.70%	
			
**Age (yrs)**			
Mean	39.7	45.7	
Median	40	46	
S. Deviation	8.3	8.3	
Min	23	24	
Max	60	61	Z = -6.37, p < 0.001
			
**Score**			
Mean	18.4	14.7	
Median	18	15	
S. Deviation	1.1	1.6	
Min	17	7	
Max	21	16	Z = -16.81, p < 0.001
Injured:			
Yes	75 (25%)	43(32%)	
No	225 (75%)	90(68%)	Chi2 = 2.5, p < 0.114
# Injuries:			
Mean	0.37	0.52	
Median	0	0	
S. Deviation	0.75	0.99	
Min	0	0	
Max	4	7	Z = -1.6, p < 0.11
**Injured & Lost Work Time**			
Yes	32 (11%)	22 (17%)	Chi2 = 2.9, p < 0.09
No	268 (89%)	111 (83%)	
			
**Rank (yrs):**			
Mean	7.6	11.4	
Median	5	10	
S. Deviation	6.6	7.8	
Min	0	0	
Max	32	31	Z = -4.7, p < 0.001
			
**Tenure (yrs)**			
Mean	12.9	18.2	
Median	11	19	
S. Deviation	8.3	9.4	
Min	1	1	
Max	35	40	Z = -5.5, p < 0.001

#### Part Two: Intervention

All injury cases were reviewed for the year before this study and the year following. ICD 9 codes were tabulated and all injury cases underwent medical review. Injuries not related to functional movement such as burns, abrasions, and lacerations were excluded from the analysis. A historical control group was formulated and compared with the intervention population.

## Results

### Part One. Functional Movement Screen

Based on simple linear regression, increasing age, rank and tenure were associated with a lower functional movement score. Each yearly increase in age resulted in a 0.1 unit decrease in overall score (p < 0.001). After adjusting for age in multiple linear regression, firefighters with a history of prior injury scored 0.24 points lower than those without history of prior injury, though this difference was not statistically significant (p = 0.25). The outcome variable was dichotomized to pass (FMS score >16) and fail (FMS score <16). Multiple logistic regression suggested that after adjusting for participant age, the odds of failing the functional movement screen were 1.68 (% confidence interval: 1.04, 2.71) times greater for firefighters with a history of any injury (p = 0.033).

### Part Two. Intervention

To test if the percent change in injuries before and after intervention was significant, a two-sample test of proportions was calculated. This test assumes under the null hypothesis that the probability of injury pre- and post-intervention are equal.

Comparing the number of injuries pre- and post-intervention of these 433 firefighters, lost time injuries were reduced by 62%, whereas total injuries were reduced by 44% compared to a historical control group. The two-sample test of proportions indicated that significant reductions were made among injuries of the back (p = 0.024) and upper extremities (p = 0.0303), however, no significant change was found for injuries of the lower extremities (p = 0.4624). Similar conclusions were reached with lost time injuries – significant reductions in both injuries to the back (p = 0.0036) and upper extremities (p = 0.0141). Results can be seen in Table 2.

**Table 2:** Intervention Summary Descriptive Statistics

433 participants	Number of injuries in historical control group	Number of injuries in intervention group	Percent Reduction (p-value*)
1. Total back, injuries	39	22	44% (0.024)
2. Total upper extremity injuries	29	15	48% (0.0303)
3. Total lower extremity injuries	10	7	30% (0.4624)
Lost time back injuries	29	11	62% (0.0036)
2. Lost time upper extremity injuries	21	8	62% (0.0141)
3. Lost time lower extremity injuries	8	3	62% (0.1292)

* Significance test estimated using a 2-sample test of proportion

## Discussion

Based on *linear *regression, there is a correlation between past musculoskeletal injury and FMS score. A history of an injury lowered the fire fighter FMS score by 3.44 (maximum of 21 points). See Table 3.

**Table 3:** Linear Regression

**Simple Linear Regression**					
**Outcome = (Overall Score - 21)**					
**Model**	**Variable**	**Coeff**	**P > |Z|**	**95% CI**	**R-square**
1	Constant	3.78	0.001	(3.57, 3.99)	
	Female	-0.74	0.093	(-1.60, 0.13)	0.007
2	Constant	-0.36	0.427	(-1.26, 0.54)	
	Age	0.099	0.001	(0.08, 0.12)	0.163
3	Constant	2.938	0.001	(2.64, 3.24)	
	Rank	0.091	0.001	(0.06, 0.12)	0.053
4	Constant	2.54	0.001	(2.18, 2.90)	
	Tenure	0.08	0.001	(0.06, 0.10)	0.120
5	Constant	2.6	0.001	(2.46, 2.75)	
	Any Injuries	3.69	0.001	(3.43, 3.95)	0.638
6	Constant	3.69	0.001	(3.46, 3.91)	
	# Injuries	0.12	0.328	(-0.12, 0.36)	0.002
7	Constant	3.7	0.001	(3.49, 3.92)	
	Injured & Lost Time	0.28	0.368	(-0.33, 0.89)	0.002
**Multiple Linear Regression**					
**Outcome = (Overall Score - 21)**					
**Final Model Only**					
**Model**	**Variable**	**Coeff**	**P > |Z|**	**95% CI**	**R-square Adjusted**

1	Constant	0.99	0.001	(0.41, 1.57)	
	Age	0.04	0.001	(0.03, 0.05)	
	Any Injuries	3.44	0.001	(3.18, 3.71)	0.661

Based on *logistic *regression, there is no significant correlation between injuries and FMS score. However, there was a significant correlation between age, rank, and tenure and FMS score as noted in Table 4.

**Table 4:** Logistic Regression

**Simple Logistic Regression**					
**Outcome = Overall Score Failure (≤ 16)**					
**Model**	**Variable**	**Count**	**OR**	**P > |Z|**	**95% CI**
1	Male	408	referent	--	--
2	Age	433	1.09	0.001	(1.06,1.12)
3	Rank	433	1.07	0.001	(1.04,1.11)
4	Tenure	433	1.07	0.001	(1.04,1.10)
5	No Injuries	315	referent	--	--
	Any Injuries	118	1.43	0.115	(0.92,2.24)
6	# Injuries	433	1.22	0.093	(0.97,1.54)
7	No Time Lost	379	referent	--	--
	Injured & Lost Time	54	1.66	0.090	(0.92,2.98)
**Multiple Logistic Regression**					
**Outcome = Overall Score Failure (≤ 16)**					
**Significant Models Only**					
**Model**	**Variable**	**OR**	**P > |Z|**	**95% CI**	**LROC**

1	Age	1.09	0.001	(1.06, 1.12)	
	Any Injuries	1.68	0.033	(1.04, 2.71)	0.703
2	Age	1.09	0.001	(1.06, 1.12)	
	# Injuries	1.29	0.044	(1.01, 1.66)	0.702
3	Age	1.09	0.001	(1.06, 1.12)	
	Injured & Lost Time	1.85	0.054	(0.99, 3.44)	0.702

NIOSH (the U.S. National Institute for Occupational Safety and Health) has advised that occupational screening programs are a priority research area. The U.S. Preventive Services Task Force has recommended specific guidelines to decide if a screening test such as FMS is effective, and whether it will improve clinical outcomes [21]. For fire fighters, an important screening component is essential---are fire fighters fit enough to safely perform the demanding physical tasks of their occupation without risk of injury?

To what degree did prior injuries hamper the subjects' ability to perform the functional movement screen tests? If a firefighter had residual physical limits from a past injury would it be logical to assume their performance would be diminished on our testing. Fortunately, all 433 firefighters complete a rigorous annual physical examination where such limitations would be noted. In addition all firefighters after an injury must be cleared to return to full unrestricted duties by the fire department occupational medicine specialist. The number of "walking wounded" --- those who were on full duty, but with undetected physical limits--- would thus be minor.

There was a significant correlation between age, rank, and tenure and FMS score.

These three variables are chronologically related and increase with time in service as a fire fighter. In general, flexibility and strength decline with age [22,23] and injuries are more likely to accumulate.

There is a correlation between past musculoskeletal injury and FMS score based on *linear *regression (An injury lowered the fire fighter FMS score by 3.44.), and there was a significant correlation between age, rank, and tenure and FMS score.

One of the major caveats to the 2-sample test of proportions in this study is the loss of power from the underutilization of paired data. McNemar's test would have been better for assessing significant differences before and after intervention, however, the paired data needed to calculate those estimates were unavailable at the time of this analysis. Still, the results of the 2-sample test of proportions should provide a relatively unbiased estimate of the before and after differences in injuries.

## Conclusion

These findings suggest that development and implementation of functional movement enhancement programs to prevent injuries in high risk workers such as firefighters is warranted.
